# Persistent Subclinical Inflammation and Long-term Functional and Cognitive Outcomes After Dengue Shock and Septic Shock in Vietnam

**DOI:** 10.1093/ofid/ofaf632

**Published:** 2025-10-08

**Authors:** Angela McBride, Nguyen Lam Vuong, Huynh Thi Le Duyen, Phan Vinh Tho, Luong Thi Hue Tai, Nuyen Thanh Phong, Nguyen Thanh Ngoc, Lam Minh Yen, Nguyen Van Hao, Sophie Yacoub, Martin J Llewelyn, Louise Thwaites

**Affiliations:** Dengue and Emerging Infection Research groups, Oxford University Clinical Research Unit, Ho Chi Minh City, Vietnam; Centre for Tropical Medicine and Global Health, University of Oxford, Oxford, UK; Dengue and Emerging Infection Research groups, Oxford University Clinical Research Unit, Ho Chi Minh City, Vietnam; Department of Medical Statistics and Informatics, University of Medicine and Pharmacy at Ho Chi Minh City, Ho Chi Minh City, Vietnam; Dengue and Emerging Infection Research groups, Oxford University Clinical Research Unit, Ho Chi Minh City, Vietnam; Hospital for Tropical Diseases, Ho Chi Minh City, Vietnam; Hospital for Tropical Diseases, Ho Chi Minh City, Vietnam; Hospital for Tropical Diseases, Ho Chi Minh City, Vietnam; Dengue and Emerging Infection Research groups, Oxford University Clinical Research Unit, Ho Chi Minh City, Vietnam; Dengue and Emerging Infection Research groups, Oxford University Clinical Research Unit, Ho Chi Minh City, Vietnam; Department of Medical Statistics and Informatics, University of Medicine and Pharmacy at Ho Chi Minh City, Ho Chi Minh City, Vietnam; Dengue and Emerging Infection Research groups, Oxford University Clinical Research Unit, Ho Chi Minh City, Vietnam; Centre for Tropical Medicine and Global Health, University of Oxford, Oxford, UK; Global Health and Infection, Brighton and Sussex Medical School, Brighton, UK; Dengue and Emerging Infection Research groups, Oxford University Clinical Research Unit, Ho Chi Minh City, Vietnam; Centre for Tropical Medicine and Global Health, University of Oxford, Oxford, UK

**Keywords:** cognitive, dengue, endothelial, functional, inflammation, sepsis, shock, Vietnam

## Abstract

**Background:**

There have been no studies reporting functional, cognitive, inflammatory or endothelial outcomes after dengue shock (DS), or septic shock (SS) in Vietnam.

**Methods:**

We conducted a prospective observational study to follow-up adult survivors of DS and SS. At hospital discharge, 1-, 3-, and 6-month follow-up, we measured health-related quality of life (EQ-5D-5L), cognitive function (Montreal Cognitive Assessment, MoCA), endothelial function (EndoPAT), and plasma biomarkers of inflammation (ferritin, IL-6, CRP) and endothelial activation (Ang1, Ang2, VCAM-1).

**Results:**

Participants included survivors of DS (n = 130), SS (n = 26), and healthy controls (n = 25). Survivors of DS had median EQ-5D-5L visual analogue score (VAS) > 90/100 at all time-points, and mildly impaired MoCA scores at hospital discharge, which had normalized by 3 months (normal ≥ 26, median [25th;75th centile] 23/30 [20;26] at discharge, 27/30 [25;29] by 3 months). Survivors of SS had lower median EQ-5D-5L VAS at all time-points (median [25th;75th centile] 80/100 [70;95] at discharge, and 90/100 [80;95] by 6 months), but MoCA scores never returned above the normal threshold (median 17/30 [13;19] at discharge, 20/30 [17;21] at 6 months). We found higher IL-6 and ferritin at all post-discharge time points in both DS and SS groups versus healthy controls (*P* < .01 for all comparisons). After 6 months, 38% with DS and 62% with SS still had ferritin levels >95th percentile of the healthy control distribution. There was little evidence of simultaneous endothelial activation.

**Conclusions:**

This is the first report of persistent subclinical inflammation after DS, and SS in Vietnam; further research is required to determine the duration and clinical significance of this phenomenon.

## BACKGROUND

Survivors of severe and critical infections frequently experience increased mortality, and physical, cognitive and psychological difficulties, which persist long after discharge from hospital [1–3]. The reasons underlying these phenomena are not clear; hypotheses include ongoing inflammation and immune dysregulation, elevated risk of recurrent infection, and accelerated cardiovascular pathology [4, 5]. Patients with elevated inflammatory markers at the time of discharge from hospital or intensive care (ICU) have higher mortality and morbidity, and poorer mobility than those who do not [6–8]. Very few studies have followed up patients after discharge to assess resolution of inflammation, but evidence is emerging that both inflammatory and endothelial biomarkers can remain elevated after apparent resolution of the precipitant infection [9, 10]. While endothelial dysfunction associated with endothelial glycocalyx degradation and low nitric oxide bioavailability is common during acute infectious shock, there have been no studies measuring endothelial function more than 2 weeks after hospital discharge [11, 12]; impaired nitric oxide–dependent vasodilation has been associated with elevated long-term cardiovascular risk [13]. Further investigation is warranted to explore whether there is evidence of persistent inflammation, endothelial activation, and/or dysfunction in adults recovering from dengue shock or septic shock.

Studies documenting post-critical care trajectories have almost exclusively been performed in high income countries (HIC), where the population are older and more comorbid than low and low-middle income countries (LMICs), and where the spectrum of infectious diseases causing admission to the ICU are different. In Vietnam, patients surviving infectious shock are not routinely followed up by healthcare professionals after discharge, and at present, there are no community rehabilitation programs. There have been no studies reporting functional or cognitive outcomes after the 2 most common causes of infectious shock in Vietnam; dengue shock and septic shock. In order to design interventions to support patients surviving critical infection, it is vital that the post-hospital health outcomes are better understood.

To address this, we performed a prospective observational study to follow-up adult survivors of dengue shock and septic shock in Vietnam. We aimed to describe the trajectories of cognitive function, biomarkers of inflammation and endothelial activation, and endothelial vasodilatory function over 6 months, and health-related quality of life (HRQol) over 12 months after discharge from hospital.

## METHODS

We conducted a prospective observational study at the Hospital for Tropical Diseases (HTD), a tertiary referral center for infectious diseases in Ho Chi Minh City (HCMC), Vietnam. We recruited participants aged ≥16 years with dengue shock between 2019 and 2022. Comparator groups included participants with septic shock and healthy controls.

Patient consent statement: We obtained ethical approval from the Oxford Tropical Research Ethics Review Committee and the Ethics review committee at HTD. All participants provided written informed consent. Participants aged 16–17 years provided written assent in addition to written consent provided by their parent/guardian.

We recruited participants within 24 hours of the diagnosis of shock. Participants underwent a series of clinical, ultrasonographic, and laboratory analyses to characterize the inflammatory and endothelial pathology underlying their circulatory failure; full details and results of these investigations are published elsewhere [14]. Diagnostic criteria for dengue shock and septic shock were in line with the World Health Organization (2009) and Sepsis 3 definitions, respectively [15, 16].

At hospital discharge, 1, 3, and 6 months later, surviving participants underwent plasma sampling for endothelial and inflammatory biomarkers. At the same timepoints, we tested endothelial nitric oxide–dependent vasodilatory function by peripheral artery tonometry using the EndoPAT device, whereby digital pulse volume changes during reactive hyperemia are measured using pneumatic finger probes to generate the reactive hyperemia index (RHI, normal ≥1.67) [17]. We conducted EQ-5D-5L HRQoL assessments and the Montreal Cognitive Assessment (MoCA) at the same timepoints, with a final EQ-5D-5L assessment by telephone at 12 months. If the participant was unable to attend an in-person follow-up appointment during prolonged COVID-19–related travel restrictions, we invited them to complete the EQ-5D-5L by telephone and omitted the MoCA, EndoPAT, and blood tests. Briefly, the MoCA is a sensitive tool for detection of mild cognitive impairment, assessing short term memory, visuospatial abilities, executive functions, attention, concentration and working memory, language, and orientation to time and place. We used the paper Vietnamese language version of the MoCA 7.1, which has an inbuilt correction for low education; if the subject has 12 years of education or less, 1 point is added to their score [18]. The EQ-5D-5L is a simple, generic measure of health for clinical and economic appraisal, comprising a descriptive questionnaire over 5 dimensions: mobility, self-care, usual activities, pain/discomfort, and anxiety/depression. Participant responses are used to generate a health state index, which can be compared against population normative data. In addition, participants complete a visual analogue score to rate their own overall health status.

### Biomarker Analysis

We refrigerated blood samples immediately after phlebotomy. C-reactive protein (CRP) was measured in real time in the hospital laboratory. Within 2 hours of phlebotomy, we centrifuged the remaining blood and stored aliquots of plasma at −80 ^o^C for later analysis. We analysed the following biomarkers of endothelial activation using a multiplexed magnetic bead-based assay on a Luminex 200 platform according to the manufacturer's specifications: angiopoietin-1 (Ang1), angiopoietin-2 (Ang2), and vascular cell adhesion molecule (VCAM1) (R&D Systems). We measured the biomarkers of inflammation, ferritin, and interleukin-6 (IL-6), using the Elecsys electrochemiluminescence immunoassay on the Cobas e 411 analyzer (Roche system).

### Healthy Controls

Since population normative data are not available in Vietnam for either the biomarkers or RHI, we recruited 25 frequency age-matched healthy controls from within the staff network at the Oxford University Clinical Research Unit, HCMC. Controls were eligible if they did not report any of the following: chronic illness, regular medications, current pregnancy, any febrile illness in the prior 28 days, or diagnosis of/treatment for dengue or bacterial infection in the previous 6 months. Healthy controls underwent phlebotomy and EndoPAT assessment at a single time-point.

### Statistical Methods

We performed statistical analysis in Stata version 17.0 (StataCorp, Texas) and R statistical software version 4.1.0. For this analysis, we defined abnormal biomarker values as those below the 5th percentile (for Ang1) or above the 95th percentile (for CRP, IL-6, ferritin, Ang2 and VCAM1) of the healthy control distribution. Taking into account multiple testing, we considered a *P*-value of <.01 as statistically significant for between group comparisons.

We summarized the distribution of the plasma biomarkers and RHI by median and interquartile range, and used the Wilcoxon Rank Sum test to compare biomarker levels between groups with dengue shock, septic shock and healthy controls. We assessed correlations between baseline characteristics (age, Charlson comorbidity index, admission SOFA score) and the biomarkers which were significantly different between either/both patient group and healthy controls during follow-up (IL-6, ferritin, and VCAM1) using plots and Spearman's correlation coefficients.

We mapped participant responses from each EQ-5D-5L subdomain to a published EQ-5D-5L value set generated by a population survey in Vietnam to generate the utility index for each combined health state (1 = health state equivalent to perfect health, <0 = health state worse than death [19]). We summarized EQ-5D-5L utility index and visual analogue scores (VAS) and MoCA results as median (25th;75th centiles) and compared between patient groups by Wilcoxon Rank Sum test.

Since the study was heavily affected by loss to in-person follow-up during the COVID-19 pandemic, we performed an exploratory analysis to investigate whether there was a difference in health status between patients who completed in-person versus telephone follow-up. We selected the VAS component of the EQ-5D-5L assessment as a proxy for health status since this measure was completed both in person and by telephone and used 2-sample T-tests to compare VAS between groups.

## RESULTS

Of participants with dengue shock, 130/135 (96%) survived to hospital discharge, and no further deaths occurred during the 12-month follow-up period. Of participants with septic shock, 26/37 (70%) survived to hospital discharge, and 4 further deaths occurred during follow-up (n = 2 at 1–3 months, n = 1 at 3–6 months, n = 1 at 6–12 months post-discharge). Families reported that 3 died from pre-existing severe liver disease, and one from “general poor health”. Overall, 12-month mortality for the septic shock cohort was 15/37 (41%). Table 1 summarizes the demographics and in-hospital clinical course of both groups; the group with dengue shock were younger, had fewer comorbidities, and required less organ support than the comparator group with septic shock. A full breakdown of the Charlson comorbidity index and body mass index categories can be found in Supplementary Table 1.

**Table 1.** Demographics, Baseline Characteristics, and Clinical Outcomes by Diagnosis

	N	Dengue Shock (N = 135)^a^	N	Septic Shock (N = 37)^a^	*P* Value^b^
Age, years	135	26 (20; 33)	37	55 (47; 63)	**<.001**
Sex, male	135	66 (43)	37	23 (62)	.194
Smoker	135	0 (0)	37	8 (22)	**<.001**
BMI (kg/m^2^)	135	22.9 (20.3; 26.3)	37	23.2 (20.8; 25.8)	.788
Charlson comorbidity index	135	…	37	…	**<.001**
0	…	122 (90)	…	10 (27)	**…**
1	…	10 (7)	…	7 (19)	**…**
2	…	0 (0)	…	5 (14)	**…**
3	…	3 (2)	…	4 (11)	**…**
4	…	0 (0)	…	2 (5)	**…**
5	…	0 (0)	…	4 (11)	**…**
6	…	0 (0)	…	4 (11)	**…**
7	…	0 (0)	…	1 (3)	**…**
Taking regular medication	135	2 (1)	37	14 (38)	**<.001**
SOFA score at enrollment	135	7 (6; 7)	37	10 (9; 13)	**<.001**
In-hospital death	135	5 (4)	37	11 (30)	**<.001**
ICU admission	135	34 (25)	37	30 (81)	**<.001**
Hospital length of stay (days)	135	5 (4; 7)	37	11 (7; 15)	**<.001**
Required invasive/non-invasive ventilation	135	13 (10)	37	19 (51)	**<.001**
Required vasopressors	135	8 (6)	37	35 (95)	**<.001**
Duration of IV fluid requirement	135	2 (2; 2)	37	4 (2; 7)	**<.001**
Required hemofiltration	135	7 (5)	37	10 (27)	**<.001**

^a^Median (25%; 75%); n (%).

^b^Wilcoxon rank sum test; Fisher’s exact test.

Due to COVID-related movement restrictions, fewer participants were able to complete in-person follow-up assessments than the EQ-5D-5L; therefore, in each table/graph, numbers completing each assessment are reported clearly. Supplementary Table 2 compares VAS scores between those who were followed up in-person versus by telephone; aside from slightly lower VAS in the in-person follow-up dengue shock group at 6 months (EQ-5D-5L VAS 94.92/100 vs 98.24/100, *P* = .0001), there were no significant differences between scores at other follow-up timepoints for either dengue shock or septic shock.

### EQ-5D-5L

**Figure 1.**
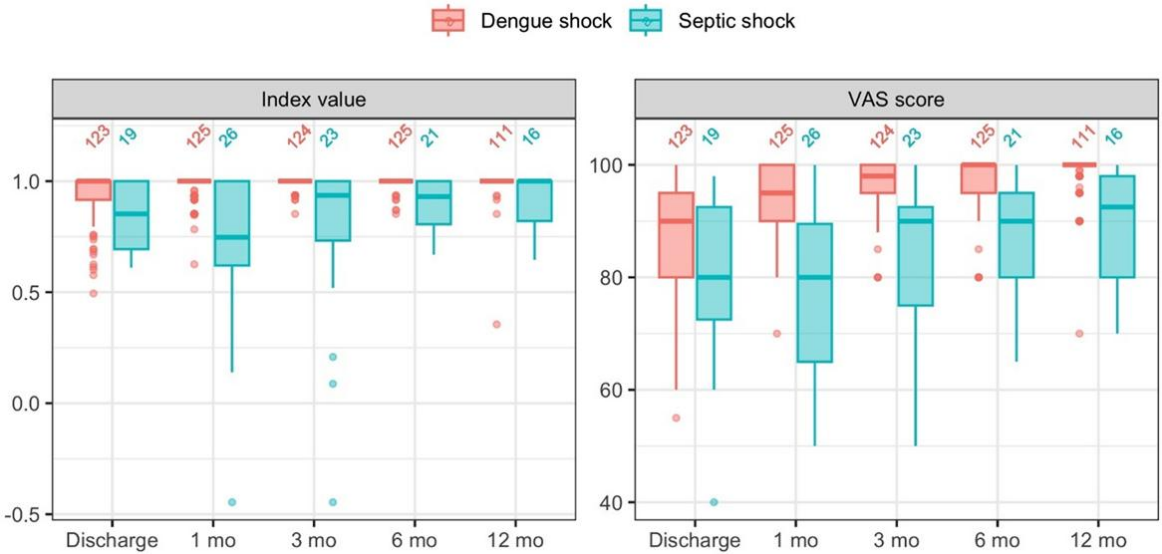
Serial EQ-5D-5L Utility index values and Visual Analogue Scores during follow-up. Numbers across the top of each chart indicate the number of patients completing each follow-up assessment. VAS, visual analogue score.

Table 2 and Figure 1 show the trajectories of the EQ-5D-5L utility index value and VAS during the follow-up period. Supplementary Figures 1 and 2 show the breakdown of EQ-5D-5L subcomponent scores for participants with dengue shock and septic shock, respectively. Participants with dengue shock had higher median EQ-5D-5L scores compared to those with septic shock throughout the follow-up period (*P* < .001 for all timepoints). Aside from one exception, EQ-5D-5L scores improved between each follow-up interval in both groups. However, for participants with septic shock, summary statistics for all outcome measures dipped between discharge and 1 month. This is due to 7 of the most unwell participants being transferred to another hospital for further management after acute treatment for septic shock (eg, surgical intervention, myocardial infarction). These participants were not available for assessment at hospital discharge but rejoined follow-up at 1 month.

**Table 2.** Serial EQ-5D-5L Utility Index and Visual Analogue Scores, and Montreal Cognitive Assessment Scores by Diagnosis

	N	Dengue Shock (N = 135)^a^	N	Septic Shock (N = 37)^a^	*P* ^b^
EQ-5D-5L utility index					
Discharge	123	1 (0.92; 1)	19	0.85 (0.69; 1)	.014
1 m	125	1 (1; 1)	26	0.75 (0.60; 1)	**<.001**
3 m	124	1 (1; 1)	23	0.94 (0.61; 1)	**<.001**
6 m	125	1 (1; 1)	21	0.93 (0.81; 1)	**<.001**
12 m	111	1 (1; 1)	16	1 (0.80; 1)	**<.001**
EQ-5D-5L visual analogue score					
Discharge	123	90 (80; 95)	19	80 (70; 95)	.076
1 m	125	95 (90; 100)	26	80 (65; 90)	**<.001**
3 m	124	98 (95; 100)	23	90 (70; 95)	**<.001**
6 m	125	100 (95; 100)	21	90 (80; 95)	**<.001**
12 m	111	100 (100; 100)	16	92.5 (80; 98)	**<.001**
Montreal cognitive assessment					
Discharge	123	23 (20; 26)	18	17 (13; 19)	**<.001**
1 m	66	26 (23; 28)	13	16 (14; 21)	**<.001**
3 m	52	27 (25; 29)	12	21 (17; 26)	**<.001**
6 m	50	28 (25; 29)	12	20 (17;21)	**<.001**

^a^Median (25%; 75%).

^b^Wilcoxon rank sum test.

### Montreal Cognitive Assessment

Table 2 shows the serial MoCA results between hospital discharge and 6-month follow-up. Supplementary Figure 3 shows the results subdivided by severity of cognitive impairment. MoCA scores were higher for participants with dengue shock versus septic shock at all assessment intervals (*P* < .001 for all timepoints). Median scores indicated evidence for cognitive dysfunction in both dengue shock and septic shock patients at hospital discharge, which improved during the follow-up phase. Median scores for participants with dengue shock returned to the normal range (≥26/30), whereas the median score for those with septic shock did not.

Although the proportion of participants reporting ≤12 years formal education was higher in the septic shock compared to the dengue shock group, this difference was not statistically significant (76.2% for septic shock vs 60.8% for dengue shock, chi2(1)= 1.84, *P* = .175). MoCA score was negatively correlated with age at all timepoints in participants with dengue shock, but not septic shock (Supplementary Figure 4). MoCA scores were not correlated with Charlson comorbidity score, except for a negative correlation at 6 months in the septic shock group (Supplementary Figure 5).

### Biomarkers of Inflammation and Endothelial Activation

**Figure 2.**
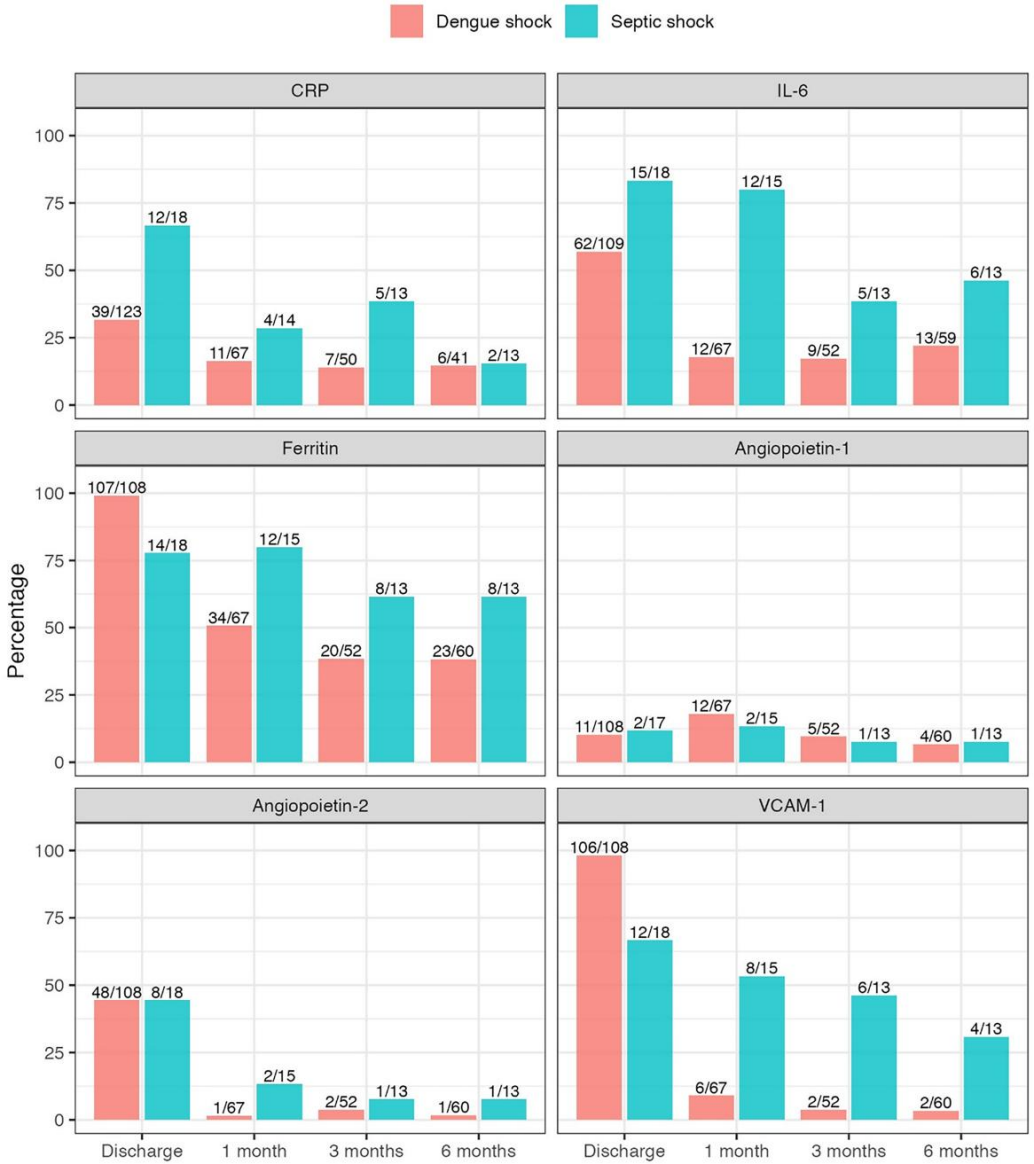
Proportion of participants with biomarker measurement >95th centile of healthy control distribution during follow-up. Ang1 < 5th centile healthy control distribution, as for this biomarker lower levels are considered abnormal, and higher levels protective.

Table 3 and Supplementary Figure 6 summarize serial biomarker results; in Supplementary Figure 6, the inpatient biomarkers (enrollment, 48 hours, discharge) have been published elsewhere, but are included here for context [14]. Figure 2 shows the proportion of participants with biomarker measurements ≥95th percentile of the healthy control distribution at each follow-up timepoint (95th percentiles as follows: CRP 3.64 mg/dl, IL-6 2.83 pg/ml, ferritin 260.4 ng/ml, Ang2 3594 pg/ml, VCAM-1 957917 pg/ml), and the proportion with Ang1 < 5th percentile of the healthy control distribution (5th percentile for Ang1 857 pg/ml).

**Table 3.** Biomarkers of Inflammation and Endothelial Activation and Reactive Hyperemia Index in Survivors of Dengue Shock, Septic Shock, and Healthy Controls

	N	Dengue Shock (N = 135)^a^	N	Septic Shock (N = 37)^a^	N	Healthy Control (N = 25)^a^	Dengue Shock Versus Septic Shock^b^	Dengue Shock Versus Healthy Control^b^	Septic Shock Versus Healthy Control^b^
CRP (mg/dl)									
Discharge	123	2.2 (1.2; 4.9)	18	7.0 (2.8; 16.1)	25	0.5 (0.3; 1.8)	**<0.001**	**<0.001**	**<0.001**
1 m	64	1.2 (0.6; 2.2)	14	2.0 (1.0; 6.3)	…	…	0.164	0.013	0.011
3 m	45	1.0 (0.5; 2.6)	13	1.1 (0.5; 3.9)	…	…	0.552	0.017	0.034
6 m	39	1.3 (0.6; 2.3)	13	1.3 (0.9; 2.7)	…	…	0.583	0.014	0.029
Ferritin (ng/ml)									
Discharge	108	1907 (1125; 3438)	18	837 (264; 1431)	25	68 (40; 121)	**<0.001**	**<0.001**	**<0.001**
1 m	67	268 (156; 603)	15	738 (3445; 1179)	…	…	**0.007**	**<0.001**	**<0.001**
3 m	52	175 (92; 332)	13	393 (175; 672)	…	…	0.015	**<0.001**	**<0.001**
6 m	60	186 (103; 320)	13	323 (137; 833)	…	…	0.129	**<0.001**	**0.001**
IL-6 (pg/ml)									
Discharge	109	3.3 (1.9; 6.7)	18	15.1 (6.9; 27.0)	25	1.5 (1.5; 1.5)	**<0.001**	**<0.001**	**<0.001**
1 m	67	1.5 (1.5; 2.36)	15	4.0 (3.6; 7.8)	…	…	**<0.001**	**0.009**	**<0.001**
3 m	52	1.5 (1.5; 2.4)	13	2.4 (1.5; 3.6)	…	…	0.248	**0.008**	**0.007**
6 m	59	1.5 (1.5; 2.5)	13	1.9 (1.5; 6.0)	…	…	0.209	**0.004**	**0.007**
Angiopoietin-1 (log2 pg/ml)									
Discharge	108	11.6 (10.8; 12.6)	17	11.8 (11.1;12.6)	25	12.0 (11.1;12.7)	0.739	0.372	0.800
1 m	67	10.8 (9.9; 12.0)	15	11.7 (10.0; 12.8)	…	…	0.536	**0.007**	0.292
3 m	52	11.8 (10.9; 12.7)	13	12.2 (11.4; 12.7)	…	…	0.632	0.750	0.716
6 m	60	12.4 (11.2; 13.2)	13	11.7 (10.7; 12.8)	…	…	0.293	0.243	0.671
Angiopoietin-2 (log2 pg/ml)									
Discharge	108	11.7 (11.3; 12.1)	18	11.7 (11.1; 12.1)	25	11.0 (10.4; 11.3)	0.886	**<0.001**	**0.003**
1 m	67	10.3 (9.9; 10.9)	15	10.7 (10.1; 11.5)	…	…	0.070	**0.004**	0.756
3 m	52	10.1 (9.7; 10.9)	13	10.9 (9.7; 11.4)	…	…	0.332	**0.003**	0.518
6 m	60	10.2 (9.5; 10.7)	13	10.4 (10.1; 11.0)	…	…	0.274	**<0.001**	0.131
VCAM-1 (log2 pg/ml)									
Discharge	108	21.4 (20.9; 22.3)	18	20.1 (19.8; 21.3)	25	19.0 (18.7; 19.2)	**<0.001**	**<0.001**	**<0.001**
1 m	67	19.1 (18.7; 19.5)	15	20.0 (19.5; 21.0)	…	…	**<0.001**	0.453	**<0.001**
3 m	52	19.0 (18.5; 19.3)	13	19.5 (18.7; 20.4)	…	…	0.037	0.541	0.085
6 m	60	18.7 (18.4; 19.1)	13	19.3 (19.1; 20.0)	…	…	**0.008**	0.115	0.085
Reactive hyperemia index									
Discharge	103	1.58 (1.33; 1.91)	26	1.50 (1.28; 1.69)	24	1.47 (1.32; 1.64)	0.445	**<0.001**	**0.005**
1 m	67	1.49 (1.31; 1.93)	14	1.67 (1.51; 1.84)	…	…	0.150	0.464	0.022
3 m	51	1.59 (1.37; 2.01)	13	1.82 (1.49; 2.19)	…	…	0.269	0.179	0.027
6 m	37	1.42 (1.28; 1.91)	13	2.06 (1.46; 2.14)	…	…	0.034	0.956	0.061

^a^Median (25%; 75%).

^b^Wilcoxon rank sum test, Bold text indicates *P* < 0.01.

Survivors of dengue shock and septic shock had persistently elevated ferritin and IL-6 at all post-discharge timepoints when compared with healthy controls (*P* < .01 for all comparisons). Six months after discharge, ferritin was greater than the 95th percentile of the healthy control distribution in 38% and 62% participants with dengue shock and septic shock, respectively. By contrast, although CRP was elevated at hospital discharge in both groups versus healthy controls, there was no difference thereafter.

With respect to biomarkers of endothelial activation, Ang1 levels were lower in survivors of dengue shock compared to healthy controls at 1-month follow-up, but this was not sustained. There were no significant differences in Ang1 levels between patients with septic shock and healthy controls after discharge. Ang2 levels were lower in those recovering from dengue shock than healthy controls at all timepoints, whereas levels for septic shock were not different to healthy controls. One month after discharge, VCAM-1 was elevated in patients with septic shock when compared to healthy controls, but no difference persisted beyond this timepoint.

The biomarkers IL-6, ferritin, and VCAM-1 were not correlated with each other at 1, 3, or 6 months post-discharge (Supplementary Figure 7). Within the dengue shock group, there was a weak positive correlation between age and ferritin, but none for age and either IL-6 or VCAM-1. Within the septic shock group, there was a positive correlation between VCAM-1 and Charlson comorbidity score at 1 and 3 month follow-up, and at the 6-month follow-up timepoint, IL-6 was positively correlated with age and negatively correlated with EQ-5D-5L VAS score (Supplementary Figures 8, 9, 11). There was no clear correlation between biomarkers during follow-up, and either Charlson comorbidity score, baseline admission SOFA score or EQ-5D-5L VAS in the dengue shock group (Supplementary Figures 9–11).

### Endothelial Function

Table 3 reports the results of serial RHI measurements. Supplementary Figure 12 presents serial RHI results graphically; the horizontal gray line indicates the published “normal” threshold (RHI ≥ 1.67). For participants with septic shock, median RHI was below the normal threshold at admission, improved by hospital discharge (11/16 participants had RHI ≥ 1.67 at discharge), and continued to improve to median 2.06 at 6 months. For participants with dengue shock, median RHI followed a similar trend toward normalization by hospital discharge, but thereafter, there was a drop in median RHI to a nadir 1.42 at 6 months follow-up. The healthy controls had unexpectedly low RHI, with median and 75th percentile of the score distribution both below 1.67 (n = 24, median [25th;75th centiles]: 1.47 (1.32–1.64).

## DISCUSSION

In this study, we followed up survivors of dengue shock and septic shock in Vietnam to understand the time course of recovery in terms of HRQoL, cognitive and endothelial function, and inflammation. Most participants reported a high HRQoL from discharge onwards, although our results indicate that some recovery is still taking place up to 12 months after discharge in those surviving septic shock. Our data suggest faster recovery of cognitive function among participants with dengue shock, and it is notable that median MoCA scores in the septic shock group remained lower than the published “normal” threshold at 6 months. Despite good functional outcomes, biomarker analysis indicated that a subset of patients with dengue shock and, to a greater extent, septic shock have prolonged subclinical inflammation, with little evidence of similarly persisting endothelial activation.

### Health-Related Quality of Life

As a reference point for our EQ-5D-5L results, a recent population study across all geographic regions of Vietnam and a variety of age groups reported a mean VAS of 81.10, and mean utility index of 0.94 (SD 0.09) [19, 20]. Patients recovering from dengue shock had EQ-5D-5L scores above these values at all time-points; those with septic shock had similar scores at discharge and above the population average between 3 and 12 months post-discharge.

This is the first study to report EQ-5D-5L after any form of severe dengue. Schulte et al [21] performed serial EQ-5D assessments after non-severe dengue; at all timepoints, patients recovering from dengue shock in our study had higher VAS than reported in their study. Regrettably, we did not formally ask participants about post-acute symptoms during follow-up; it is not clear whether the EQ-5D-5L instrument is sufficiently sensitive to detect the impact of mild symptoms, such as fatigue, difficulty concentrating and alopecia described frequently by patients recovering from dengue [22].

There are no studies reporting EQ-5D-5L results following recovery from septic shock in Vietnam, but studies performed in post-sepsis populations outside of Vietnam, mostly in HIC, found that patients report substantially lower HR-QoL than our cohort [23]. One notable exception is a study following sepsis survivors in Malawi, which reported utility scores of 0.91 by 12 weeks post-discharge (similar to the 0.94 in our dataset) [24]. Reasons for particularly favorable outcomes in LIC/LMIC may relate to a younger, less comorbid population, together with differential criteria for selection of potential ICU candidates where resources are particularly constrained. There may also be cultural and contextual differences in perception of HR-QoL, which limit direct comparison of the outcome assessment between populations. Regardless, the results reinforce that it is not appropriate to extrapolate findings regarding sepsis survivorship from HIC to LMIC, where the characteristics of the patient population, causative pathogens, and resources available for critical care and rehabilitation are very different [25].

### Cognitive Function

This is the first study to provide a detailed report on cognitive function in patients surviving dengue shock. Patients with non-severe dengue in Mexico had higher scores than our cohort during the acute phase of illness (MoCA 23 in our cohort vs 27 in Mexican cohort) [26]; lower scores in our population may reflect greater disease severity, and/or the impact of treatments or hospitalization on cognitive function.

In contrast to the temporary cognitive impairment seen in dengue shock, MoCA scores for patients with septic shock did not return to the normal range. However, this cohort was significantly older and had more comorbidities, and although the comparison did not reach statistical significance, the proportion with ≤12 years education was higher than in the dengue shock group. Although it has been translated to Vietnamese, no validation studies could be found for the MoCA in Vietnam, and the MoCA Basic, which is designed for patients with very limited/no formal education has not been translated to Vietnamese. Nonetheless, the results do mirror a pattern described by studies in HIC, whereby sepsis-induced cognitive impairment is common, and only partially reversible [3, 27]. The mechanism underlying cognitive deterioration is thought to be multifactorial, including: cerebral hypoperfusion, ischemia, and disruption of the blood brain barrier with ingress of immune factors, damage-associated and pathogen-associated molecular patterns [27, 28]. One theory for the reversible element of cognitive dysfunction involves heparan sulfate fragments, cleaved from the endothelial glycocalyx during acute shock, crossing the leaky blood–brain barrier and temporarily blocking BDNF-mediated hippocampal signaling [29, 30]. If true, this mechanism may also explain a degree of the temporary cognitive impairment seen after dengue shock.

### Biomarkers of Inflammation and Endothelial Activation

We have shown that some survivors of dengue shock, and to a greater extent septic shock, have persistently elevated IL-6 and ferritin during the 6 months after discharge from hospital when compared with healthy controls; these results raise the possibility of persisting rather than resolving pathology in a subset of survivors, although pre-existing inflammation cannot be ruled out by our study design. Inflammatory biomarkers were not correlated with comorbidity burden, baseline disease severity, or self-reported HR-QoL after discharge.

This is the first study to explore whether persistent inflammation occurs after clinical recovery from dengue shock, but evidence is emerging that survivors of sepsis and COVID-19 may have persistent inflammation, which is associated with adverse clinical outcomes in some cases [9, 10, 31]. Although this study was not powered to investigate long-term outcomes for patients with persistent inflammation after infection, it is clear from the wider literature that even low-level inflammation is not a benign state. Subclinical inflammation is associated with cognitive decline [32], lower self-reported HR-QoL [33], and increased mortality [34–36]. Indeed, CRP and IL-6 have emerged as even stronger cardiovascular risk predictors than traditional biomarkers such as LDL cholesterol [37–39], and several trials have now demonstrated the benefit of immune modulation in patients with low-level persistent inflammation, independent of serum lipid modification [38, 40]. Our preliminary findings highlight the need for definitive studies to determine the long-term trajectory of post-infectious inflammation, and the impact, if any, on clinical outcomes.

Despite the evidence for persistent inflammation, biomarkers of endothelial activation reverted toward the distribution of healthy controls rapidly after discharge. The only exception was VCAM1, whose distribution was elevated in survivors of septic shock versus healthy controls at 1 month (54%), 3 months (46%), and 6 months (31%). Yende et al [10] also found that 23% of sepsis survivors had at least one elevated VCAM1 level in the first year after hospital discharge; however, their study reported an even greater loss to follow-up study than ours (only 8.7% of all patients completed the post-discharge schedule of 3 blood samples).

### Endothelial Function

The RHI has previously been measured in research participants recovering from acute dengue virus infection. Although study follow-up ended at 2 weeks post-discharge, the authors found a similar trend to that seen in our study; RHI was initially low, improved by hospital discharge, and down-trended thereafter [41]. Our finding replicates and extends this finding; for patients with dengue shock, follow-up RHI was consistently lower than values recorded at hospital discharge. It is unclear why participants with dengue shock, but not septic shock had low RHI after discharge, considering the latter group had more comorbidities, which are associated with impaired endothelial function. Furthermore, the healthy control RHI data are difficult to explain; scores were much lower than those reported from large studies in populations outside Vietnam. One potential explanation is that most follow-up time-points for dengue shock patients, and the healthy control testing was done during the dry season in HCMC, when air quality is typically very poor, whereas follow-up for septic shock survivors was a year-round activity. Short-term exposure to traffic related air pollution has been linked to impaired RHI, but without controlling for the air quality index on sampling days, this theory cannot be tested [42].

### Strengths and Limitations

This is the first study to report post-acute outcomes for patients with dengue shock and for septic shock in Vietnam. While our findings begin to fill an important knowledge gap, the major limitation is sample size; with loss to in-person follow-up during the COVID-19 pandemic, the number of patients completing all assessments is too small to draw firm conclusions on the frequency, duration and clinical impact of post-infectious inflammation in either patient group. Further, since the patients were recruited at the time of shock onset, we cannot rule out that they did not have subclinical inflammation preceding, or even predisposing them to, their incident infection. Comparisons drawn on HrQoL and cognitive function between patients with dengue shock and septic shock should be interpreted with caution, as these patient groups had different demographics, and may have had differential pre-morbid functional status. Ideally, these shortcomings would be evaluated in a prospective cohort with baseline functional assessments, biomarker measurements, and a longer follow-up duration, but since dengue shock and septic shock are rare outcomes, the number of patient years follow-up required to achieve sufficient endpoints may not be feasible.

## CONCLUSIONS

We have shown that survivors of dengue shock and septic shock in Vietnam broadly report good functional outcomes after ICU discharge. Despite this, we demonstrate evidence that a proportion experience prolonged subclinical inflammation persisting beyond symptomatic recovery. In our cohort, persistent inflammation was not accompanied by evidence of prolonged endothelial activation. The duration of the phenomenon, and its impact on long-term health outcomes should be investigated in a dedicated study.

## Supplementary Material

ofaf632_Supplementary_Data

## Supplementary Data

Supplementary materials are available at *Open Forum Infectious Diseases* online. Consisting of data provided by the authors to benefit the reader, the posted materials are not copyedited and are the sole responsibility of the authors, so questions or comments should be addressed to the corresponding author.
