# Supplementary material for: Persistent Subclinical Inflammation and Long-term Functional and Cognitive Outcomes After Dengue Shock and Septic Shock in Vietnam
Source: Open Forum Infect Dis. 2025 Oct 8;12(11):ofaf632. doi: 10.1093/ofid/ofaf632 (PMC12575078; doi:10.1093/ofid/ofaf632)
Supplement: ofaf632_Supplementary_Data [file ofaf632_supplementary_data.docx]

**Supplementary Table 1. Body mass index and Charlson comorbidity scores**

|  | n | Dengue shock  (N=135)^1^ | n | Septic shock  (N=37)^1^ |
| --- | --- | --- | --- | --- |
| **Body mass index** |  |  |  |  |
| BMI (kg/m^2^) | 135 | 22.9 (20.3; 26.3) | 37 | 23.2 (20.8; 25.8) |
| BMI category (Asian cut-off) | 135 |  | 37 |  |
| Acceptable |  | 68 (50) |  | 18 (49) |
| Overweight |  | 43 (32) |  | 14 (38) |
| Obese |  | 24 (18) |  | 5 (14) |
| **Charlson comorbidity index** | 135 |  | 37 |  |
| 0 |  | 122 (90) |  | 10 (27) |
| 1 |  | 10 (7) |  | 7 (19) |
| 2 |  | 0 (0) |  | 5 (14) |
| 3 |  | 3 (2) |  | 4 (11) |
| 4 |  | 0 (0) |  | 2 (5) |
| 5 |  | 0 (0) |  | 4 (11) |
| 6 |  | 0 (0) |  | 4 (11) |
| 7 |  | 0 (0) |  | 1 (3) |
| **Charlson Comorbidity sub-components** |  |  |  |  |
| Hypertension | 135 | 4 (3) | 37 | 11 (30) |
| Angina on minimal exertion | 135 | 0 (0) | 37 | 0 (0) |
| Prior myocardial infarction | 135 | 0 (0) | 37 | 1 (3) |
| Grade I-III Heart failure | 135 | 0 (0) | 37 | 0 (0) |
| Grade IV Heart failure | 135 | 0 (0) | 37 | 0 (0) |
| Peripheral vascular disease | 135 | 0 (0) | 37 | 0 (0) |
| Anemia | 135 | 0 (0) | 37 | 1 (3) |
| Cerebrovascular disease | 135 | 0 (0) | 37 | 0 (0) |
| Hemiparesis | 135 | 0 (0) | 37 | 0 (0) |
| Chronic obstructive pulmonary disease | 135 | 0 (0) | 37 | 0 (0) |
| Severe respiratory disease | 135 | 0 (0) | 37 | 1 (3) |
| Connective tissue disease | 135 | 0 (0) | 37 | 0 (0) |
| Peptic ulcer disease | 135 | 3 (2) | 37 | 0 (0) |
| Mild liver disease | 135 | 2 (1) | 37 | 2 (5) |
| Moderate/severe liver disease | 135 | 1 (1) | 37 | 7 (19) |
| Moderate/severe kidney disease | 135 | 1 (1) | 37 | 1 (3) |
| Diabetes | 135 | 3 (2) | 37 | 8 (22) |
| Diabetes with complications | 135 | 0 (0) | 37 | 0 (0) |
| Dementia | 135 | 0 (0) | 37 | 0 (0) |
| Metastatic solid tumor | 135 | 0 (0) | 37 | 0 (0) |
| Haematological malignancy | 135 | 0 (0) | 37 | 1 (3) |
| Elective surgery within 3 months | 135 | 0 (0) | 37 | 0 (0) |
| AIDS | 135 | 0 (0) | 37 | 1 (3) |
| Immunocompromised | 135 | 0 (0) | 37 | 0 (0) |

^1^Median (25^th^ centile, 75^th^ centile); n (%)

**Supplementary Table 2. Comparison of Visual Analogue Scores for patients who attended in-person versus telephone follow-up**

|  | n | In-person  VAS score^1^ | n | Telephone  VAS score^1^ | P^2^ |
| --- | --- | --- | --- | --- | --- |
| Dengue shock |  |  |  |  |  |
| 1 month | 66 | 94.15 (6.60) | 59 | 95.57 (5.22) | 0.1869 |
| 3 months | 52 | 96.23 (4.35) | 72 | 96.63 (4.80) | 0.6281 |
| 6 months | 60 | 94.92 (5.87) | 65 | 98.24 (3.10) | **0.0001** |
| Septic shock |  |  |  |  |  |
| 1 month | 13 | 78.46 (15.33) | 13 | 75.61 (15.25) | 0.6393 |
| 3 months | 12 | 86.58 (15.16) | 11 | 78.18 (14.19) | 0.1855 |
| 6 months | 12 | 88.50 (9.01) | 9 | 84.22 (13.75) | 0.3944 |

*^1^mean (SD), ^2^2 sample t test*

**Supplementary Figure 1. Serial EQ-5D-5L subcomponent scores for participants with dengue shock**

**
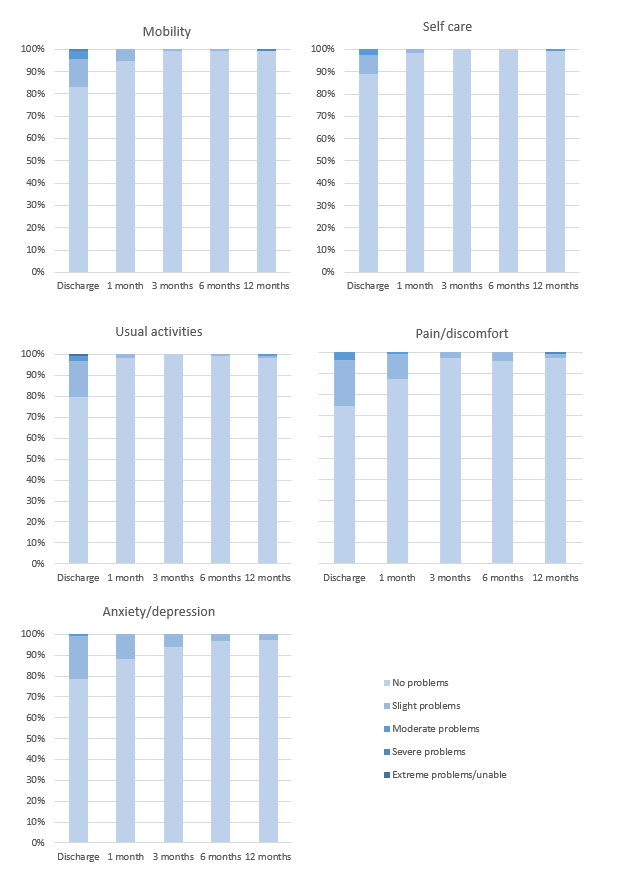
**

**Supplementary Figure 2. Serial EQ5D subcomponent scores for participants with septic shock**

**
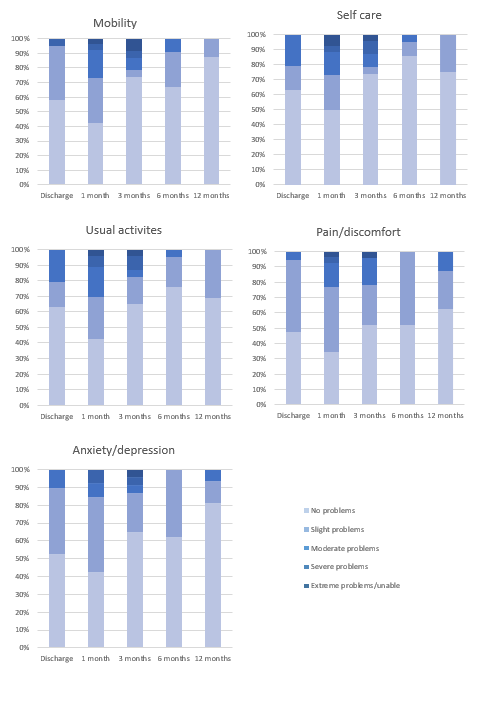
Supplementary Figure 3. Serial MoCA scores for participants during the follow-up period subdivided by severity of cognitive impairment**


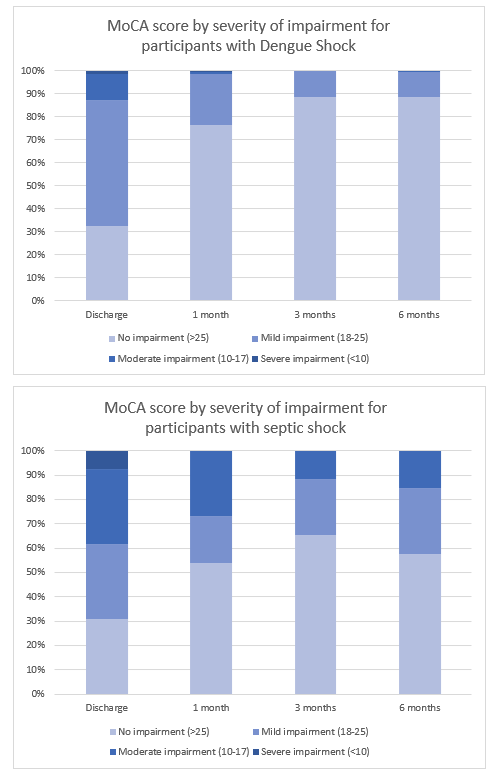


**Supplementary Figure 4. Association between age and Montreal Cognitive Assessment (MoCA) scores after discharge**


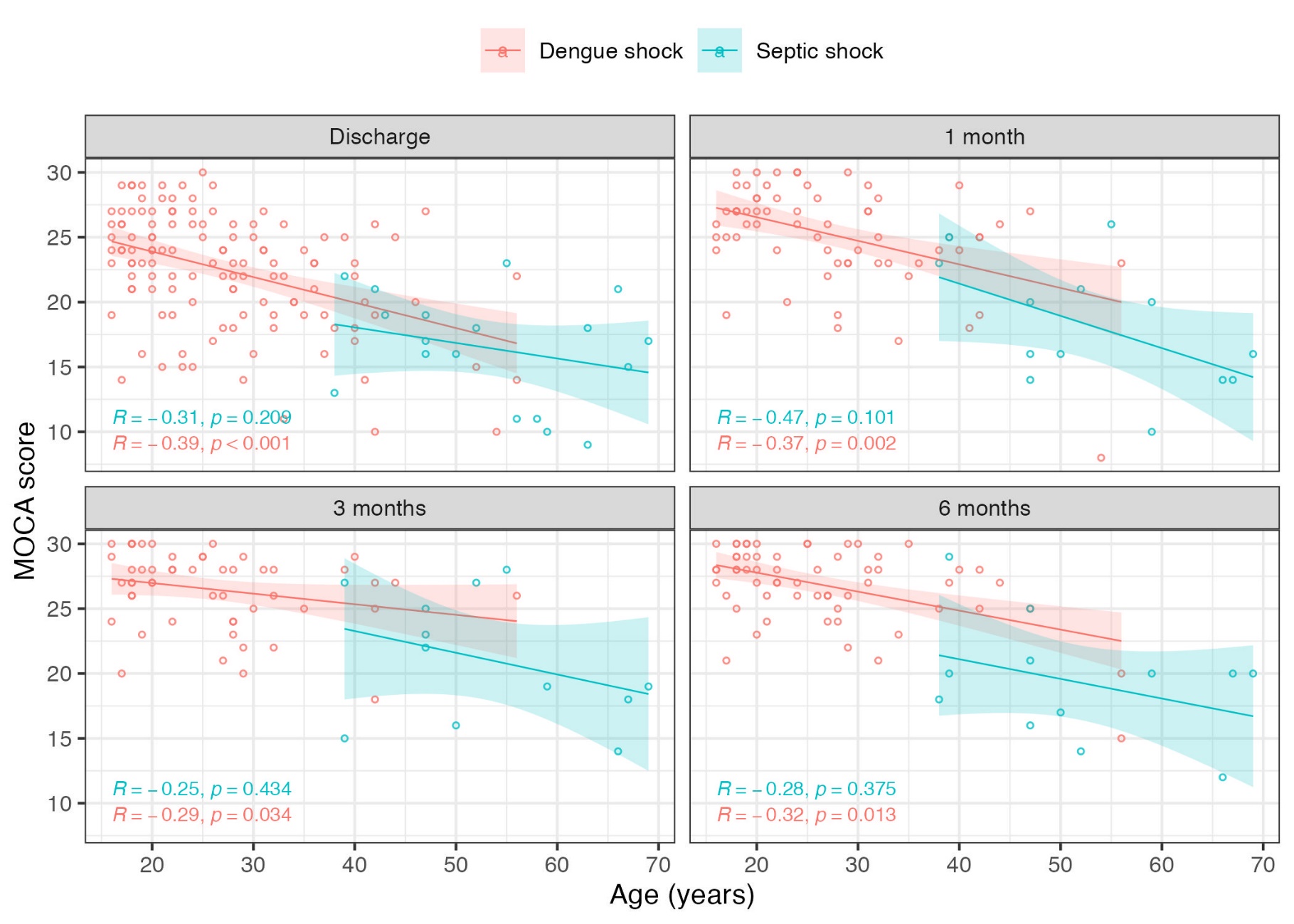


**Supplementary figure 5. Association between Charlson Comorbidity score and Montreal Cognitive Assessment (MoCA) score after discharge**


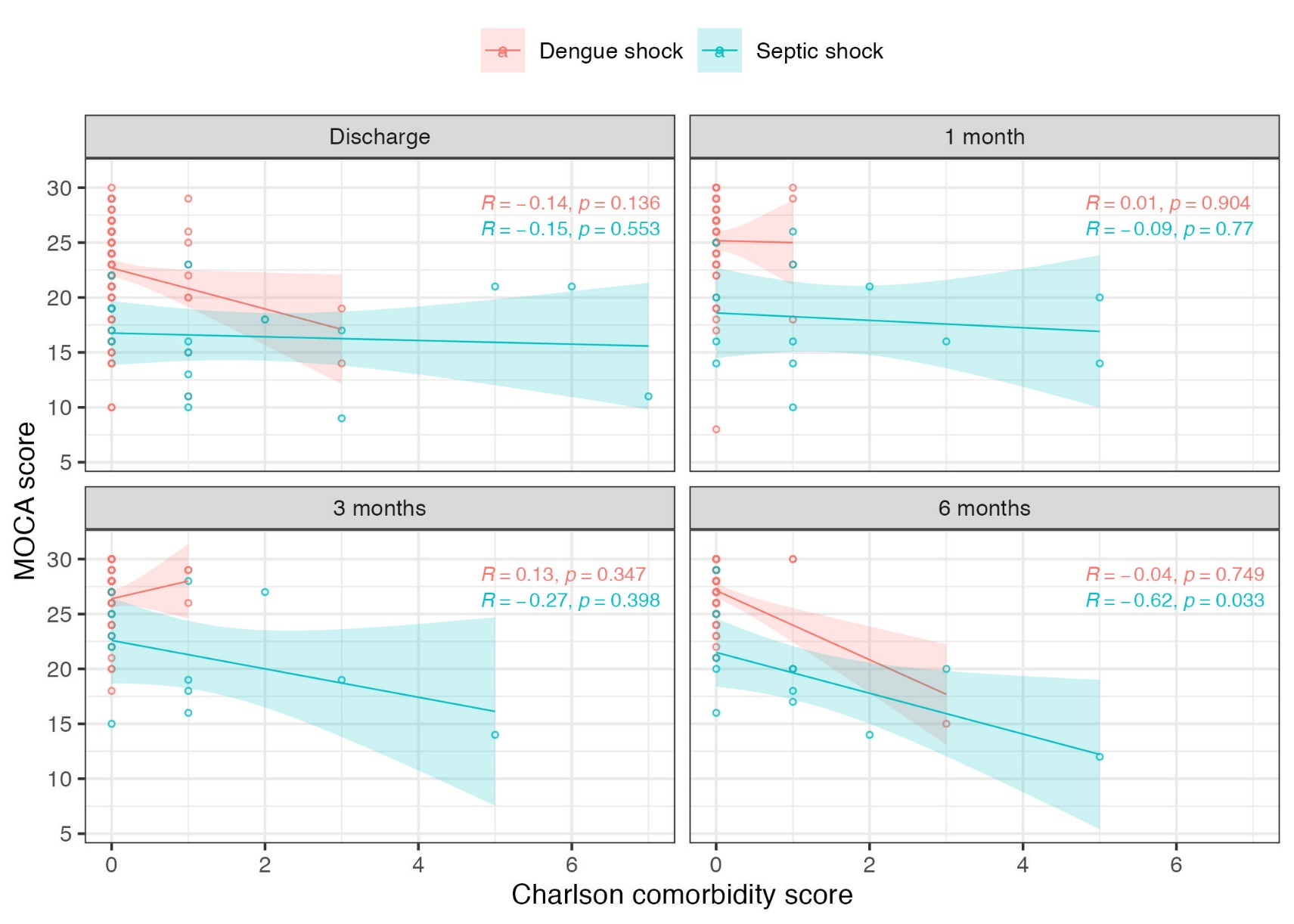


**Supplementary figure 6. Serial Inflammatory and endothelial biomarkers during the study follow-up period**


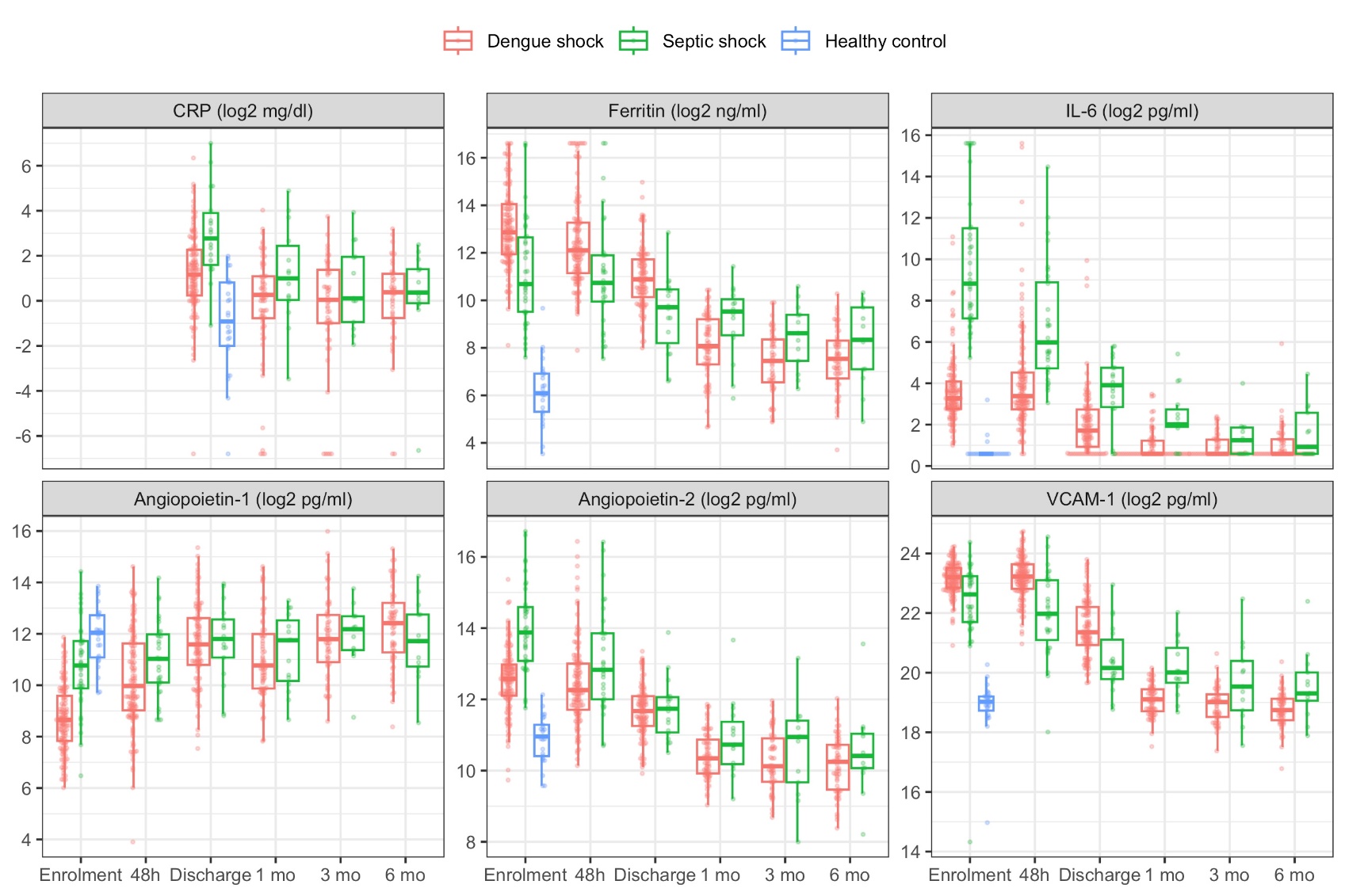


**Supplementary figure 7. Association between IL-6, ferritin and VCAM-1 after discharge**

**
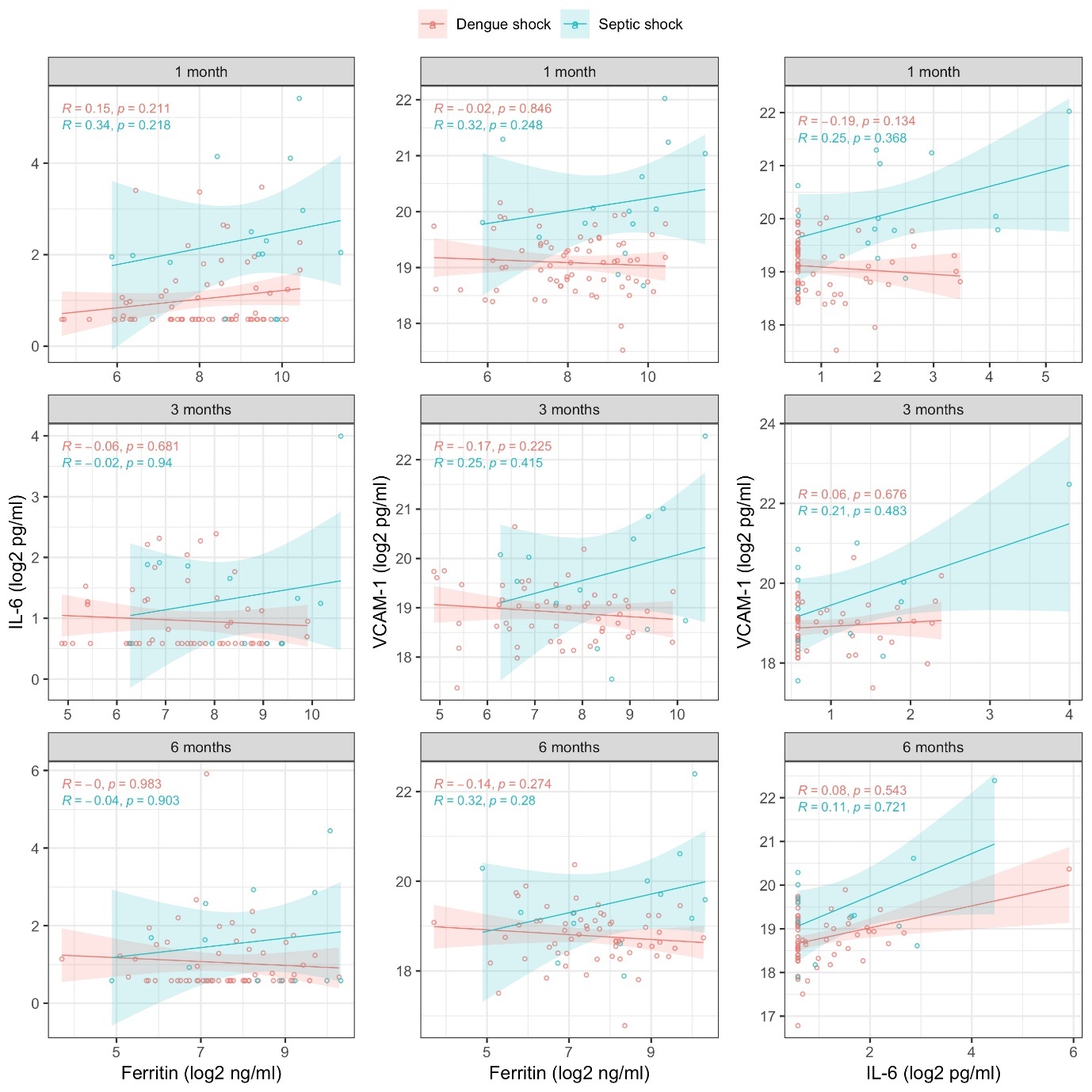
**

**Supplementary figure 8. Association between age and IL-6, ferritin and VCAM1 after discharge**


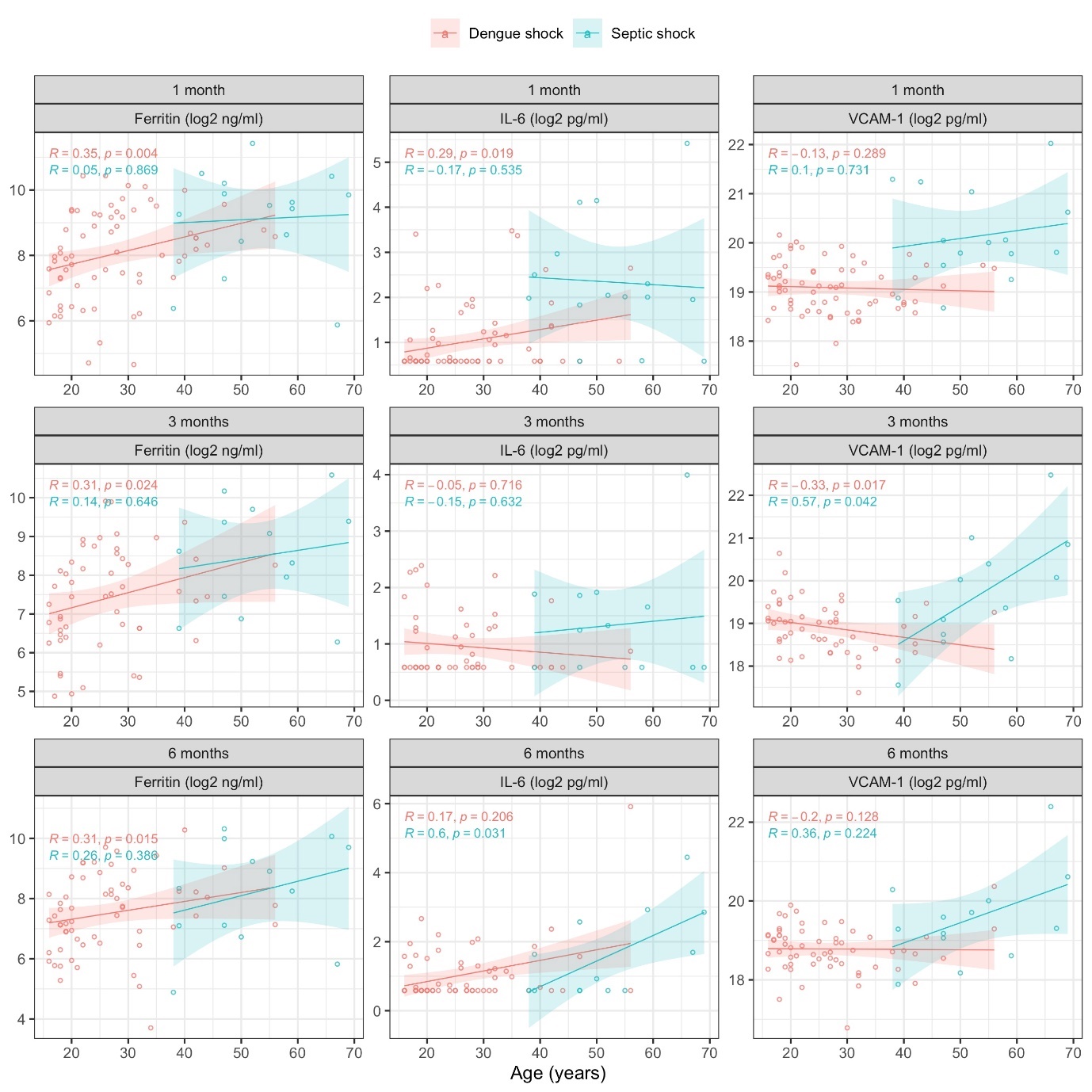


**Supplementary figure 9. Association between Charlson comorbidity score and IL-6, ferritin and VCAM-1 after discharge**


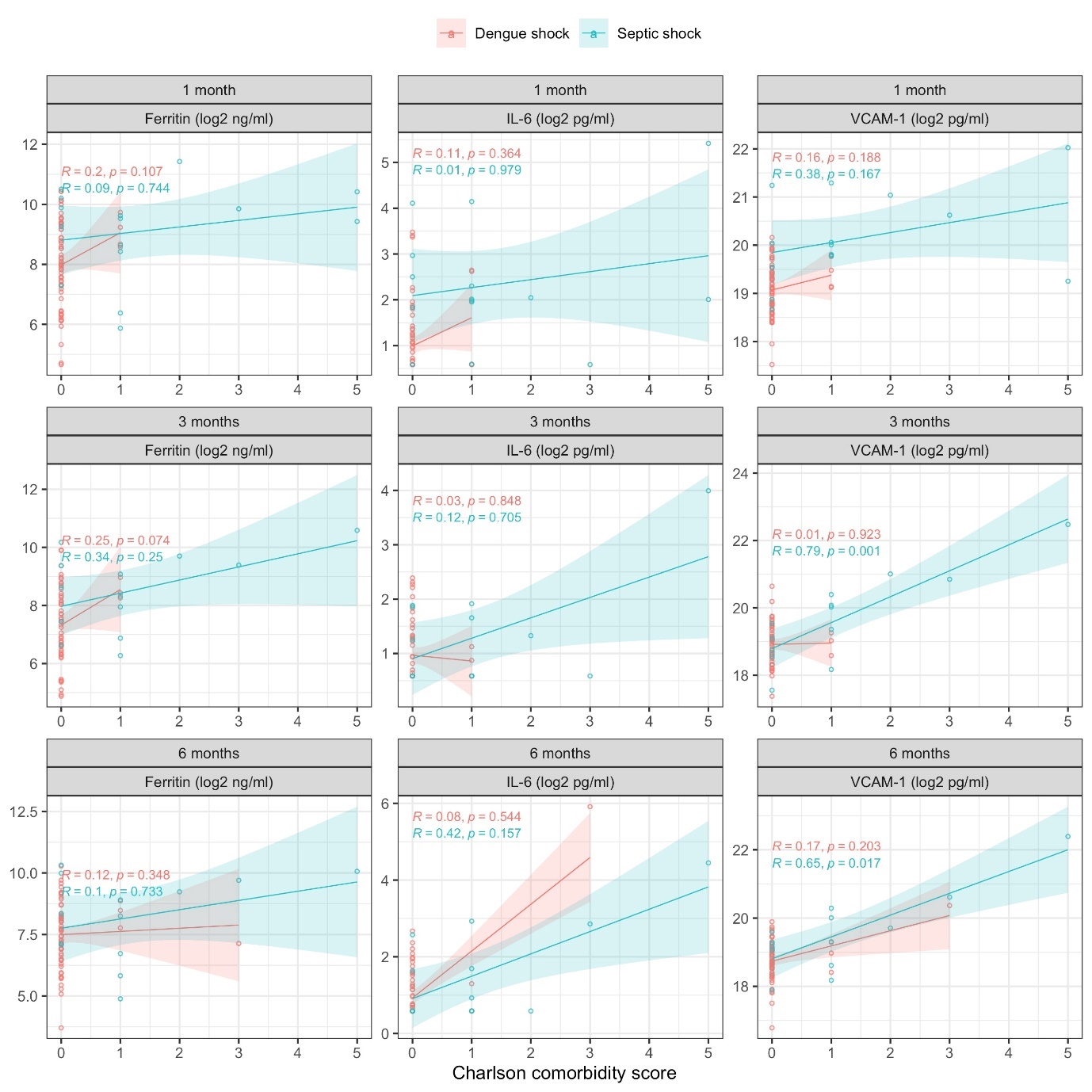


**Supplementary figure 10. Association between admission Sequential Organ Failure Assessment score and IL-6, ferritin and VCAM-1 after discharge**


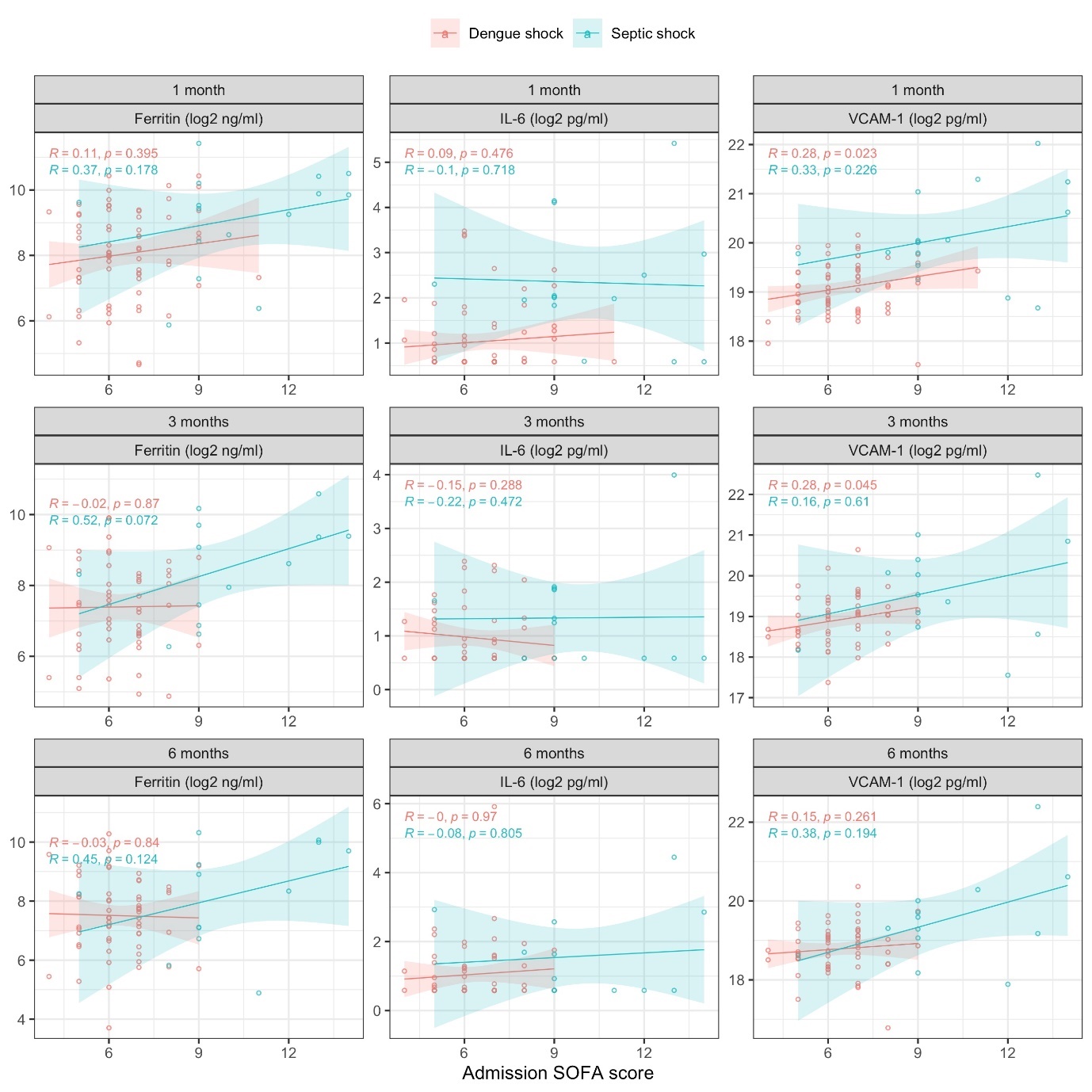


**Supplementary figure 11. Association between EQ-5D-5L Visual analogue score and IL-6, ferritin and VCAM1 after discharge**


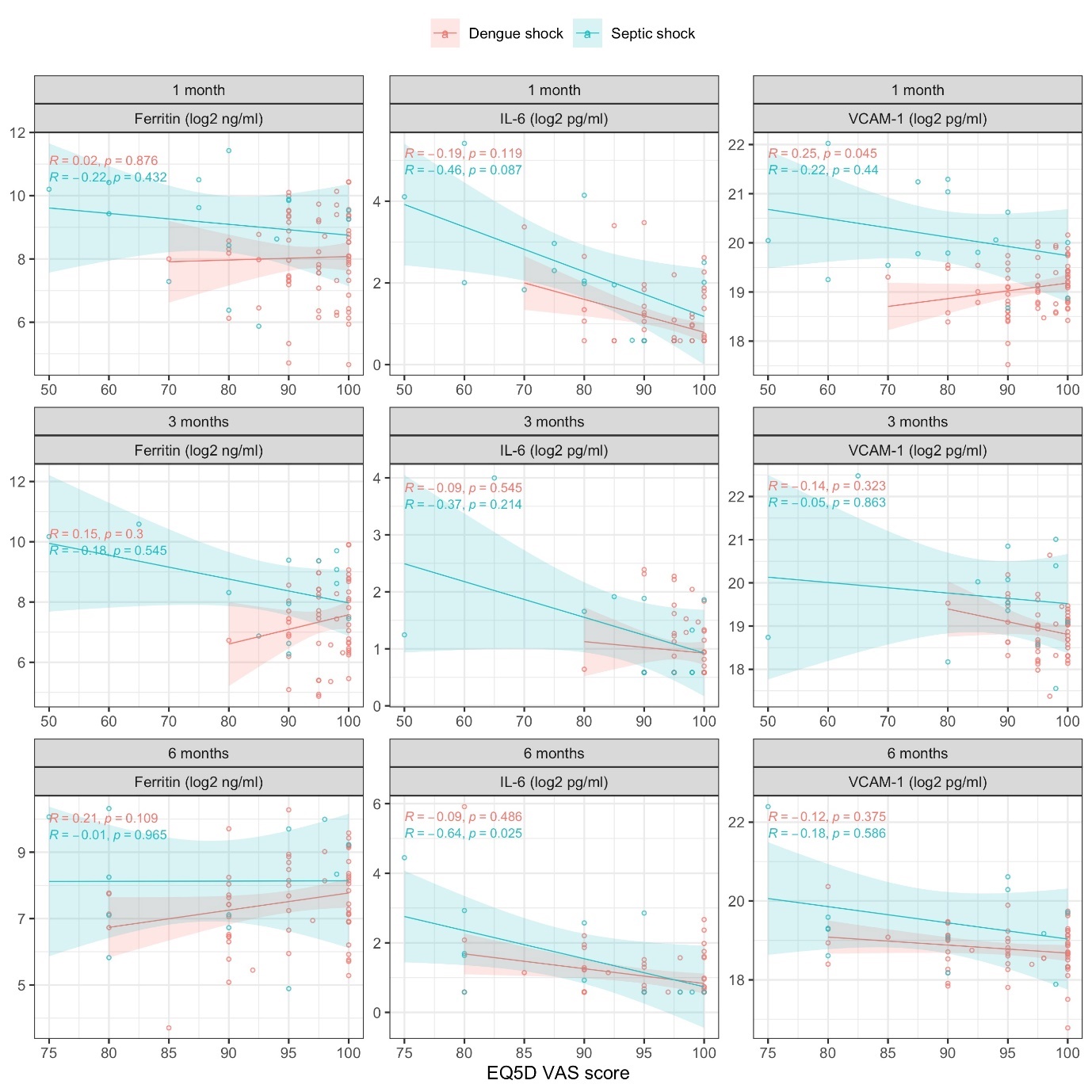


**Supplementary Figure 12. Serial Reactive hyperaemia index in participants with Dengue shock and Septic shock**

**
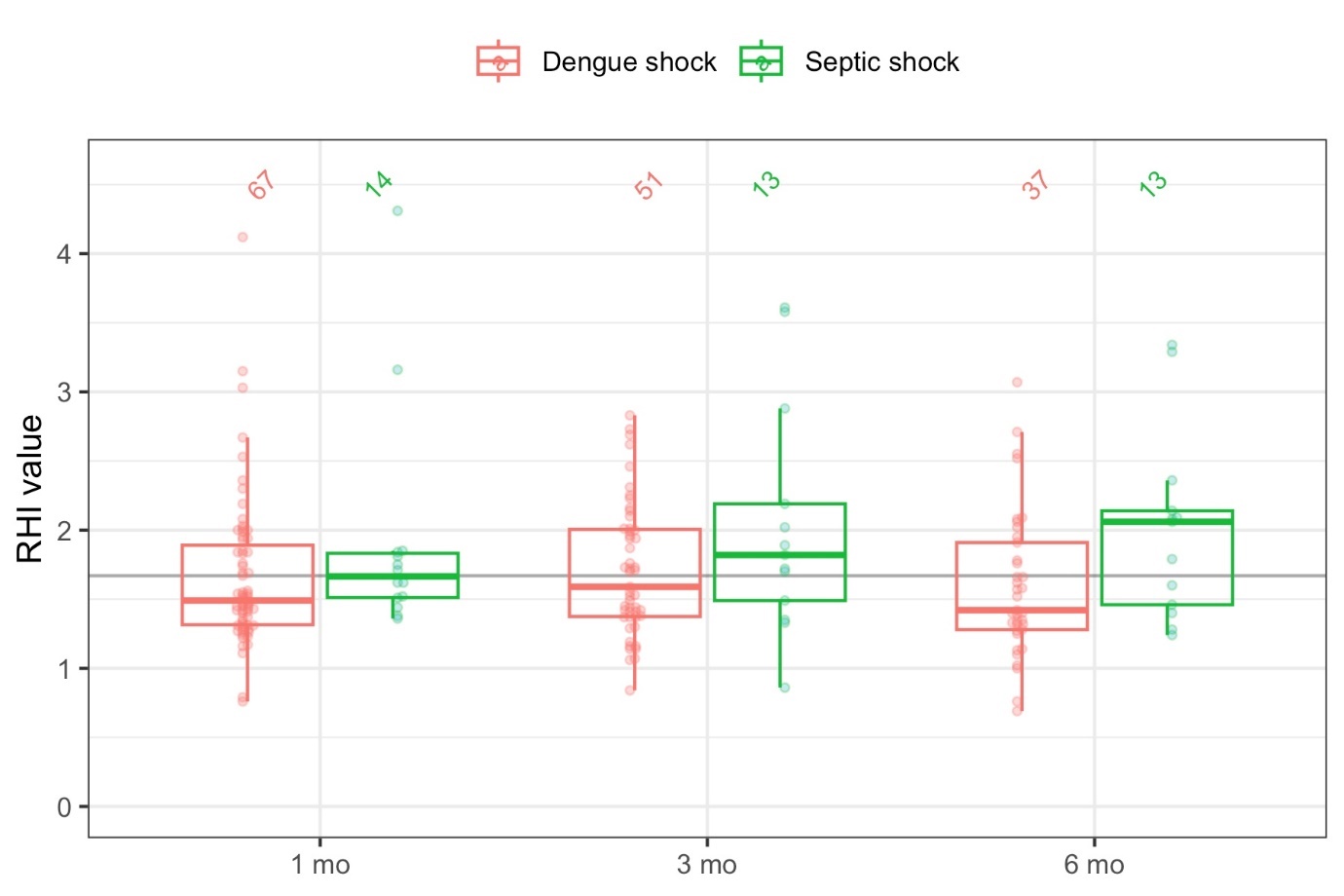
**

*The numbers above the box-and-whisker plots indicate the number of participants at each follow-up interval. RHI = reactive hyperaemia index, mo=months*
